# Formation of Lipid and Protein Oxidation Products during In Vitro Gastrointestinal Digestion of Dry-Cured Loins with Different Contents of Nitrate/Nitrite Added

**DOI:** 10.3390/foods10081748

**Published:** 2021-07-29

**Authors:** Guadalupe Lavado, Nieves Higuero, Manuel León-Camacho, Ramón Cava

**Affiliations:** 1 Tradinnoval Research Group, INBIO G+C, University of Extremadura, 10003 Cáceres, Spain; glavador@alumnos.unex.es (G.L.); nhiguero@alumnos.unex.es (N.H.); 2 Lipid Characterization and Quality Department, Instituto de la Grasa, Spanish National Research Council, 41012 Seville, Spain; mLeon@ig.csic.es

**Keywords:** nitrite, nitrate, in vitro digestion, dry-cured loin, conjugated dienes, malondialdehyde, carbonyls, thiols

## Abstract

The effect of nitrate/nitrite (0, 37.5, 75, and 150 mg/kg) in the dry-cured loin formulation on the formation of lipid and protein oxidation products during in vitro digestion was evaluated. Dry-cured loins formulated with nitrate/nitrite resulted in significantly less lipid and protein oxidation than uncured loins before and after simulated digestion. Compared to loins added with 0 mg/kg nitrate/nitrite, dry-cured loins with 37.5, 75, and 150 mg/kg contained a significantly lower content of conjugated dienes, malondialdehyde, carbonyls, and non-heme iron, and higher amounts of nitrosylmioglobin and thiols. During in vitro digestion, the content of conjugated dienes, malondialdehyde, and carbonyls increased, while thiol content decreased, indicating the development of lipid and protein oxidative processes. At the end of the intestinal phase, the 75 mg/kg digests had a significantly higher content of conjugated dienes, while no differences were found among the other digests. During the in vitro intestinal phase (180 and 240 min), nitrate/nitrite curing resulted in significantly lower malondialdehyde concentrations in the 37.5, 75, and 150 mg/kg loin digests than in the uncured loin digests. No significant differences were observed at the end of the intestinal digestion phase between the cured loin digests. Digests of dried loins without nitrate/nitrite addition showed higher carbonyl contents than the nitrate/nitrite cured counterparts. The loss of thiols was significantly higher in loin digests without added nitrate/nitrite than in loin digests with different amounts of curing salts. The addition of 37.5 mg/kg nitrate/nitrite in the cured loin formulation prevents the formation of lipid peroxidation products and carbonyls from protein oxidation and thiol loss during digestion

## 1. Introduction

Dry-cured meat products are formulated with nitrite (NO_2_^−^) in conjunction, or not, with nitrate (NO_3_^−^) as curing agents. Sodium nitrite is widely used in dry-cured meat products to develop a characteristic color via the formation of nitrosylmyoglobin NOFe(II)Mb (NOMb) to generate a distinctive flavor, to inhibit the outgrowth of *Clostridium botulinum* and other food pathogens, and to delay oxidative deterioration [1]. The antioxidant activity of nitrite is related to a kind of cooperative mechanism that includes the formation NOMb, which has antioxidant properties per se, the chelation of non-heme iron, which forms a stable complex preventing the development of the Fenton reaction, and the stabilization of unsaturated lipids [2].

Several scientific papers and reports show that nitrite, together with certain amines or amides, can lead to the generation of the two main N-nitroso compounds (NOCs), namely nitrosamines and nitrosamides, during the cooking, smoking, or drying of muscle foods. Animal models have demonstrated NOCs to be the most potent and broadly acting carcinogens known [3,4,5,6]. In vivo, NOC can occur through both exogenous and endogenous sources. Endogenous NOCs are generated in several ways, including bacterial, cell-mediated, and acid-catalyzed nitrosation. This last method takes place in the stomach, favored by an acidic pH (pH 3.0), in which HNO_2_ derived from nitrite can be the cause of the nitrosation of secondary amines or amides [7]. Instead, nitrite can also react with both the iron in myoglobin and the free thiol groups. The reaction of nitrite with iron gives rise to the formation of nitrosylheme, which releases NO and can lead to the production of nitrosamines. Instead, the reaction of nitrite with the free thiol groups gives rise to the generation of nitrosothiols which, after digestion, can transfer their NO groups to the iron of myoglobin, giving rise to the formation of nitrosylheme [5,8]. A few years ago, the International Agency for Research on Cancer (IARC) published a report linking the ingestion of nitrates or nitrites with the possible occurrence of cancer in humans [9]. These findings have generated a reason for concern about the consumption of cured meat and the probability of developing cancer in consumers [5,6,9].

During the digestive process, muscle foods are exposed to different environments (oral, gastric, and intestine lumen) that remodel oxidative processes. Saliva presents a series of antioxidant compounds (i.e., thiocyanate, nitrite), although these are not enough to control all the oxidation processes that can take place during digestion [10]. The food bolus in the stomach finds a pro-oxidant medium due to its acidic pH and dissolved oxygen [11], while in the small intestine, the food bolus is emulsified due to the digestive enzymes causing a series of oxidation reactions [12]. During digestion, lipid oxidation products (LOP), such as malondialdehyde (MDA), 4-hydroxy-hexenal (4-HHE), or 4-hydroxy-nonenal (4-HNE), and protein oxidation products, such as carbonyls and oxidized forms of amino acids, are produced. Higher levels of these compounds are reached in the intestinal phase than are in the initial food, demonstrating that gastrointestinal digestion promotes lipid and protein oxidation [13,14,15].

Recently, van Hecke et al. [16,17,18] examined the factors dependent on the type of meat, concerning its iron content, total fat content, and the presence of nitrites/ascorbates in the generation of lipid and protein oxidation-derived compounds during the enzymatic phase of simulated digestion, and the formation of toxic compounds during the colonic fermentation of muscle foods. These authors established the interconnection of oxidizing lipids, protein oxidation, and protein nitration during the digestion of muscle foods. In simulated digested chicken, pork, and beef models, iron and total fat contents induced higher concentrations of the toxic lipid oxidation products MDA, 4-HNE, and other volatile aldehydes in the duodenal and colonic fluids, together with increased protein carbonyl compounds from protein oxidation. The increased total fat and heme-Fe content significantly favors the formation of higher concentrations of the NOC-specific DNA adduct O^6^-carboxymethyl-guanine (O^6^-C-MeG) during the colonic fermentation [16,17]. Conversely, the presence of nitrite in pork, poultry, and beef inhibited lipid oxidation and decreased protein carbonylation after in vitro gastrointestinal digestion, while an inconsistent effect on the formation of O^6^-C-MeG has been reported. Authors demonstrated that heme-Fe is not solely responsible for oxidation and nitrosation reactions throughout an in vitro digestion approach, but its effect is promoted by a higher fat content in meat [16].

The question remains whether a further reduction of residual nitrite in processed meats will benefit consumer health since the balance of •NO: ROS determines the anti- or pro-oxidant outcome of nitrite. Nitric oxide (NO) formed through nitrite added to products can act as a pro-oxidant if it is in the same proportion as ROS (1:1), while it acts as an antioxidant if it is in a higher proportion [19].

Given the above, there is a trend to reduce ingoing nitrite levels motivated by the fact that consumers demand healthier foods with fewer additives added. In response to this demand, the meat industry has started manufacturing meat products without chemical preservatives [20]. The traditional cured meat products industry is not outside of these changes and needs to adapt meat product formulation to the new consumption trends.

In this study, dry-cured loins formulated with four levels of ingoing amounts of nitrate/nitrite (0, 37.5, 75, and 150 mg/kg) were studied for the formation of lipid and protein oxidation-derived compounds during in vitro oral, gastric, and intestinal digestion. Conjugated dienes and malondialdehyde were analyzed as markers for lipid oxidation and carbonyls and thiols for protein oxidation.

## 2. Materials and Methods

### 2.1. Chemical Reagents and Equipment

The chemical reagents used for the different analyses: KCl, KH_2_PO_4_, NaHCO_3_, NaCl, MgCl_2_(H_2_O)_6_, (NH_4_)_2_CO_3_, NaOH, HCl, KOH, BK_3_O_3_, and CaCl_2_(H_2_O)_2_ were acquired from Fisher Scientific, Waltham, MA, USA. N-(1-Naphthyl) ethylenediamine dihydrochloride (NED), fluorescamine, 2,4-dinitrophenylhydrazine (DNPH), 5-5′-dithiobis (2-nitrobenzoic acid) (DTNB), 2-(N-morpholino) ethanesulfonic acid buffer (MES), guanidine hydrochloride, trichloroacetic acid (TCA), sodium dodecyl sulfate (SDS), 2-thiobarbituric acid (TBA), 1,1,3,3-tetraethoxypropane (TEP), and a Pierce Rapid Gold BCA Protein Assay Kit were purchased from Fisher Scientific, Waltham, MA, USA. 2-propanol, n-hexane, methanol, ethyl acetate, and ethanol were acquired from Fisher Scientific, Waltham, MA, USA.

α-amylase from human saliva (A1031), pepsin from porcine gastric mucosa (P6887), pancreatin from porcine pancreas (P7545), and bile extract porcine (B8631) were acquired from Merck (Darmstadt, Germany).

Assays were developed in polypropylene plastic tubes. Assays were performed under a controlled temperature using a thermomixer (Eppendorf AG, Hamburg, Germany), provided with a heated lid to avoid condensation in tube caps. A liquid handling workstation Epmotion 5073 (Eppendorf, Hamburg, Germany) was used for transferring samples to 96-well microplates prior to spectrophotometric or fluorometric readings. A microplate reader spectrophotometer Multiskan Go (Thermo Fisher Scientific, Vanta, Finland) was used for absorbance measurements. A Varioskan LUX Multimode Microplate Reader (Thermo Fisher Scientific, Vanta, Finland) was used for fluorescence readings. All the assays were run in duplicate and readings in quadruplicate.

### 2.2. Dry-Cured Loin Samples

Iberian cured loins were manufactured under commercial conditions. Four different batches were produced with decreasing ingoing concentrations of nitrate/nitrite (NOx) added: 1. 150 mg/kg NO_2_Na + 150 mg/Kg NO_3_K (150 NOx), 2. 75 mg/kg NO_2_Na + 75 mg/kg NO_3_K (75 NOx), 3. 37.5 mg/kg NO_2_Na + 37.5 mg/kg NO_3_K (37.5 NOx), and 4. no NO_2_^−^/NO_3_^−^ added (0 mg/kg NO_2_Na/NO_3_K) (0 NOx). A detailed description of the process of production is described in Higuero et al. [21].

### 2.3. In Vitro Gastrointestinal Digestion

The in vitro gastrointestinal digestions consisted of enzymatic digestion, including three sequential steps: oral, gastric, and intestinal. For the simulated digestion, the standardized static COST INFOGEST-based SGD protocol was followed [22].

Stock solutions, simulated salivary fluid (SSF), simulated gastric fluid (SGF), and simulated intestinal fluid (SIF) were prepared in the same molarity as reported in Table 1. All solutions were prepared daily and pre-warmed at 37 °C before use. Additionally, salivary α-amylase (oral phase) was prepared in SSF to a final concentration of 75 U/mL, pepsin (gastric phase) in SGF to a final concentration of 2000 U/mL, as well as pancreatin (small intestine phase) in SIF to a final concentration of 100 U/mL (based on trypsin activity). Bile salts (10 mM) were prepared in SIF.

Equal amounts of five dry-cured loins of the same batch were minced using a Premax manual mincer. Dry-cured loin samples (5 g) were sequentially incubated for 2 min with 5 mL SSF (pH 7.0), 120 min with 10 mL SGF (pH 3.0), and 120 min with 20 mL SIF (pH 7.0).

In the oral phase, 5 g of dry-cured loin were transferred to a 50 mL beaker and mixed thoroughly with 4 mL SSF solution, 0.5 mL of salivary α-amylase solution, 25 μL of 0.3 mol/L CaCl2 and 475 μL of distilled water. Then, the obtained mixture was incubated in a water bath for 2 min at 37 °C. Gastric digestion was continued by immediate addition of 8 mL of SGF, 5 μL of 0.3 mol/L CaCl_2_, and 1.03 mL of distilled water to the oral bolus, and pH was adjusted to 3.0 with 0.47 mL volume of 6 M HCl. Next, 0.5 mL of porcine pepsin was added and continuously kept under shaking (120 rpm) at 37 °C for 2 h. Then, intestinal digestion was followed by the addition of 8.5 mL of SIF, 40 μL of 0.3 M CaCl_2_ and 2.5 mL of bile salts (25 mg/mL) to the mixture. After adjusting the pH to 7 with 2.7 mL of 1 M NaOH, 5 mL of a pancreatin solution and 1.26 mL distilled water were added and kept under agitation (120 rpm) at 37 °C for 2 h. Blank samples containing only simulated fluids were prepared. Enzymatic incubations were performed in quintuplicate.

### 2.4. Sampling

**Figure 1:**
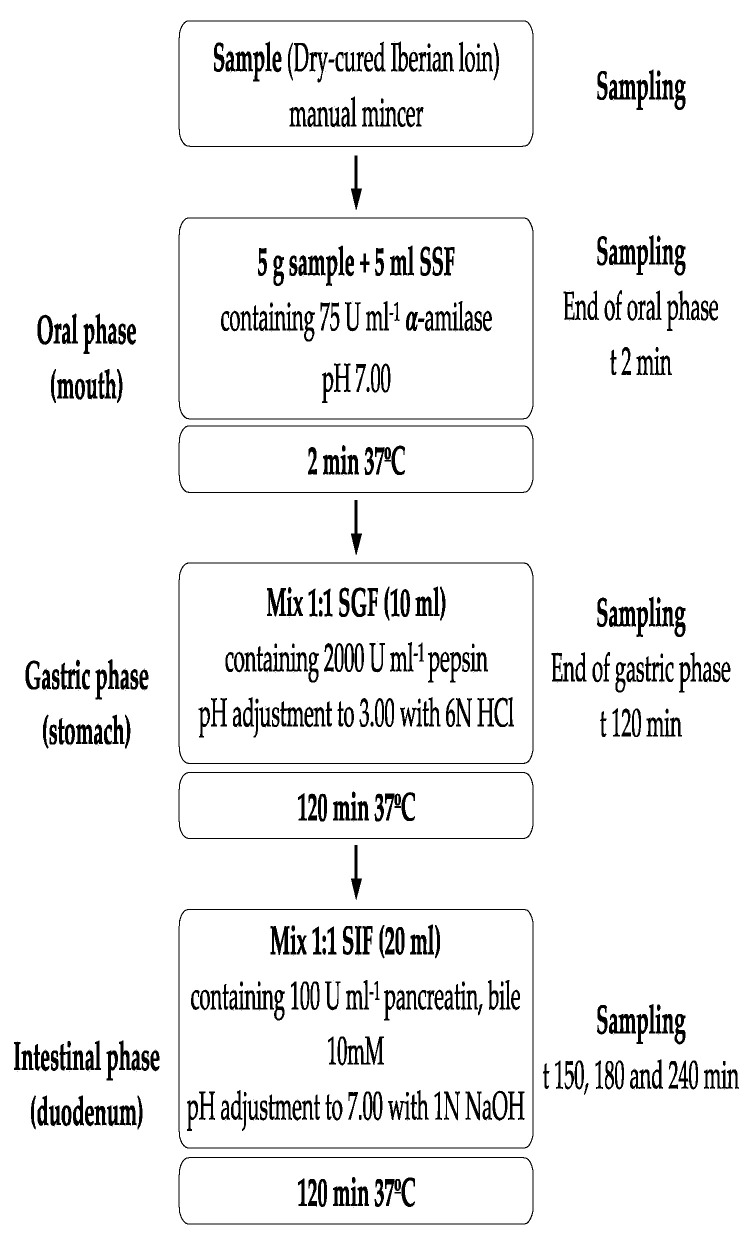
Flow diagram of the INFOGEST in vitro digestion method and sampling times in the experiment. Acronyms: SSF: Simulated Salivary Fluid, SGF: Simulated Gastric Fluid, and SIF: Simulated Intestinal Fluid. Enzyme activities are in units per mL of final digestion mixture at each corresponding digestion phase.

Samplings of digests were collected at the end of the oral (t = 2 min) and gastric phase (t = 120 min) and in the intestinal phase at 30, 60 and 120 min (t = 150, 180, and 240 min) (Figure 1) and immediately stored at −80 °C. To mimicking the liquid/solid ratio in the digested samples in the oral, gastric, and intestinal phases, the digestion volume was 35 mL using the appropriate fluids (SSF, GSF, and SIF). Thus, after completion of the oral phase, 10 mL of SGF and 20 mL of SIF were added, and 20 mL SIF was added at the end of the gastric phase [17]. Digested samples were stored at −80 °C until determinations were carried out.

### 2.5. Analytical Methods

#### 2.5.1. Nitrite Content

Residual nitrite amounts were determined by following the standard spectrophotometric method [23]. In dry-cured loins, 5 g of sample and 150 mL of 40% ethanol were gently stirred (60 °C, 30 min) and filtered. For nitrite determination, 0.5 mL of the ethanolic extract or digests was added in a test tube, followed by 0.25 mL of 0.35 mM sulfamide solution and 0.25 mL of 2 mM N-(1-Naphthyl) ethylenediamine dihydrochloride (NED) solution. After 15 min of incubation at room temperature, samples were disposed in 96-microtiter wells (x4) and the absorbance of samples was read at 538 nm using a spectrophotometer. Nitrite contents were calculated from a NaNO_2_ standard curve (0.16—0.001 mM) and results are expressed as mg/100 g of sample or nmol/mL of digest. Determinations were performed in duplicate.

#### 2.5.2. Non-Protein Nitrogen

Non-protein nitrogen in dry-cured loin and digest samples was determined fluorometrically using fluorescamine as fluorophore [24]. Dry-cured Iberian loin samples were placed for 1 h in cold water (0.7 g in 7 mL) to facilitate subsequent grinding. Extracts from dry-cured samples were prepared by homogenization with a Heidolph homogenizers SilentCrusher (Heidolph NA LLC, Schwabach, Alemania) for 30 s at 15,000 rpm.

For dry-cured loin extracts or digest samples, 150 μL aliquots were then removed and diluted with 600 μL of 12.5% trichloroacetic acid to precipitate proteins. Samples were shaken for 15 min at 4 °C. After centrifuging at 2000× *g* for 10 min, the concentration of peptides and amino acids was measured in the supernatant. First, 300 μL of the supernatant was neutralized with 300 μL of 2 M sodium borate, pH 10. Secondly, 180 μL of fluorescamine at a concentration of 0.6 mg/mL in acetone was added. Fluorescence was measured 1 h after adding fluorescamine using a Varioskan LUX Multimode Microplate Reader. A volume of 200 μL was placed in each well of black 96-well microtiter plates. Analyses were performed λ _excitation_ = 375 nm and λ _emission_ = 475 nm with excitation and emission slit setting to 10 nm. The non-specific fluorescence of corresponding fluorescamine-untreated samples was subtracted. The level of free amino groups in dry-cured loin extracts or digest samples was determined by reference to a calibration curve of glycine (5–50 mM) treated under the same conditions and at the same time as the dry-cured loin extracts or digest samples. Results are expressed as g Gly equiv/100 g of sample or mg Gly equiv/mL of digest.

#### 2.5.3. Nitrosylmyoglobin (NOMb) Content

The NOMb content determination was conducted using the spectrophotometric method described by Hornsey [25] with slight modifications. Samples (1 g) were homogenized (6.0 m/s; 60 s, 2 cycles) in 10 mL of solution (acetone: water 80:20, *v*/*v*) in a 15 mL tube that contained a lysing matrix type A (MP Biomedicals Inc., Santa Ana, CA, USA) using a Fast Prep-24TM 5G (MP Biomedicals Inc., Santa Ana, CA, USA). The homogenate was stirred using a tube roller at 4 °C for 5 min in the dark and subsequently centrifuged (5000 rpm, 4 °C, 5 min). The absorbance of the supernatant was read at 540 nm using a Shimadzu UV-1800 spectrophotometer (Shimadzu, Kyoto, Japan). NOMb concentration was calculated as NOMb (mg/kg acid hematin) = Abs_540nm_ × 290. Results are expressed as mg/100g.

#### 2.5.4. Zn-protoporphyrin (ZnPP) Content

The ZnPP content was determined by fluorescence spectroscopy based on the method described by Bou et al. [26] with some modifications. Samples (2 g) were homogenized twice (4.0 m/s; 30 s) in a 15 mL tube with 10 mL of ethyl acetate/acetic acid solvent mixture (4:1, *v*/*v*) and a lysing matrix type A (MP Biomedicals Inc., Santa Ana, CA, USA) using a Fast Prep-24TM 5G (MP Biomedicals Inc., Santa Ana, CA, USA). Both extracts and the sample residue were mixed using a tube roller in the dark (60 rpm, 4 °C, 20 min), subsequently centrifuged (1100× *g*, 14 min, 4 °C), and the supernatants filtered through a filter paper (grade 601, Fisherbrand, Thermo Fisher Scientific, Waltham, MA, USA). A Varioskan LUX Multimode Microplate Reader (Thermo Fisher Scientific, Vanta, Finland) was used for fluorescence readings. Supernatants (200 μL) were dispensed in black 96-well microtiter plates and incubated at 30 °C for 2 min. Zinc-protoporphyrin was detected by fluorescence with excitation and emission wavelengths set at 415 and 590 nm, respectively. Ethyl acetate/acetic acid solvent mixture (4:1, *v*/*v*) was used as a blank. The ZnPP content was calculated from a standard curve of ZnPP (4.00–0.0079 mg/L) and contents are expressed as mg/100 g. Readings were made in duplicate.

#### 2.5.5. Non-Heme Iron

Non-heme iron (NHI) content was determined spectrophotometrically [27]. One gram of minced product was placed in a 50 mL polypropylene tube and 10 mL of an iron extraction solution (0.05% hydroxylamine, 10% hydrochloric acid, 10% trichloroacetic acid) was added. Extraction was performed over 20 h at room temperature and the tubes were regularly stirred. The slurry was filtered through filter paper and the filtered extract used for NHI determination. On milliliter of the extract was mixed with 3 mL of the chromogen solution (0.03% bathophenanthroline sulphonic acid in 3 M sodium acetate). Absorbance was measured at 540 nm. The NHI contents were calculated from an iron standard curve (5–0.08 μg/mL). Results are expressed as mg/100 g. Extractions and determinations were assessed in duplicate for each sample.

#### 2.5.6. Lipid Extraction and Fatty Acid Profile Determination

Lipids were extracted with n-hexane (1:10 *w*/*w*) and evaporated to dryness with nitrogen. Fatty acid methyl esters (FAMEs) were analyzed by gas chromatography (GC). FAMEs were extracted with n-hexane after cold methylation with 2 N KOH in methanol, following the official method (B.O.E., 2004). GC was performed with a Varian 3900 apparatus (Varian Co, Palo Alto, CA, USA) using a fused silica capillary HP 88 column (100 m × 0.25 mm, 0.25 μm film thickness). The oven temperature was kept at 175 °C for 10 min and was then raised to 205 °C at a rate of 3.0 °C/min and held isothermally for 10.0 min. The injector temperature was kept at 240 °C, while the detector temperature was set at 250 °C. Hydrogen was used as the carrier gas at a constant head pressure of 131 KPa and a split ratio of 1:65. Air and hydrogen with flow rates of 300 and 30 mL/min, respectively, were used for the detector, which had an auxiliary flow of 30 mL/min of nitrogen [28]. Identification of fatty acids in the samples was carried out comparing retention times for standards and samples. The thirteen fatty acids were used as chemical descriptors and their peak areas as analytical signal. The quantification of individual fatty acids was carried out by evaluating the corresponding relative percentage according to the normalization area procedure, assuming an equal factor response for any species.

#### 2.5.7. Conjugated Dienes Content

The formation of conjugated dienes was monitored as increases in the absorbance at 233 nm of hexane:isopropanol (3:1) dilution of loin extracts or digests [29]. The original method was slightly modified to adapt it to samples and digest. Dry-cured loin samples (1 g) were extracted with 7 mL SSF. Loin extracts and digest samples (75 μL) were extracted with 750 mL of hexane:isopropanol (3:1) for 1 min on a vortex and then centrifuged at 4000× *g* for 5 min. A blank with distilled deionized water was similarly treated. The absorbance at 233 nm was used for the determination of conjugated diene, using a molar extinction coefficient of 25,200 M^−1^ cm^−1^. Results were expressed as µmol/100 g or µmol/mL of digest.

#### 2.5.8. Malondialdehyde Content

The extent of lipid oxidation was estimated as TBARS (thiobarbituric acid reactive substances) formed from the reaction of malondialdehyde (MDA) with 2-thiobarbituric acid under acid conditions [30]. Loin samples (2.5 g) were extracted with 7.5 mL of 3.86% perchloric acid and 250 µL of BHT. In a polypropylene test tube, a volume of homogenate or digest was mixed with an equal volume of thiobarbituric acid 0.2 M in acetic acid and heated at 90 °C for 30 min. Next, tubes were centrifuged at 5000× *g* for 5 min and supernatants were dispensed in 96-well microplates using a liquid handling workstation. Absorbance readings were measured at 532 nm in a microplate spectrophotometer. Measurements were assessed in duplicate for each sample. The MDA content was calculated from a standard curve with 1,1,3,3-tetramethoxypropane (TEP) (0.16—0.001 mM). Results were expressed as µmol/100 g of sample or µmol/mL of digest.

#### 2.5.9. Protein Carbonyl Content

Protein oxidation was estimated by measuring the carbonyl content [31]. Dry-cured loin samples (1 g) were homogenized (10,000 rpm, 40 s) with 10 mL of 0.15 M KCl. In a polypropylene test tube, 200 µL of homogenate or digest were mixed with 1 mL of 10% trichloroacetic acid (TCA). Two aliquots of each sample were used for the assay. This mixture was incubated at 4 °C for 15 min. Subsequently, the samples were centrifuged (2000× *g*, 30 min) and the supernatant was removed. The pellet was mixed with 1 mL of 10% TCA and the aforementioned process repeated. Supernatants were discarded and pellets were mixed with 500 µL of 2 M HCl and with 500 µL of 10 mM DNPH in 2 M HCl, respectively. Tubes were shaken in the dark (350 rpm, 60 min), and then 500 µL of 20% TCA was added. After 15 min on ice, the tubes were centrifugated (2000× *g*, 20 min) and supernatants discarded. Pellets were washed (×3) with 1 mL of ethanol:ethyl acetate (1:1 *v*/*v*), centrifuged (2000× *g*, 20 min), and supernatants discarded. After washes, samples were dried at 40 °C for 15 min. Subsequently, 1 mL of 6 M guanidine was added. Samples were shaken in the dark (350 rpm, 30 min) and centrifuged (9500× *g*, 10 min) before absorbance measured. Carbonyl groups were quantified by treatment with 2,4-dinitrophenylhydrazine (DNPH) to form a stable protein 2,4-dinitrophenyl hydrazone product. The carbonyl concentration was quantified spectrophotometrically at 370 nm using a molar absorption coefficient of ε hydrazone of 22.0 mM^−1^ cm^−1^. Protein concentration was measured spectrophotometrically at 280 nm after reaction with 2 M HCl instead of DNPH. For calculation of the carbonyl content (nmol/mg protein) the absorbance at 280 nm and 370 nm of the samples were used in the Equation (1) [32]:

Carbonyls = Abs_370nm_/[ε hydrazone _370 nm_ x (Abs _280nm_ − Abs _370 nm_) × 0.43] × 10^6^ (1)

Assays were performed in duplicate. Results were expressed as nmol carbonyls/mg protein.

#### 2.5.10. Thiol Content

The thiol group concentration was determined upon derivatization with Ellman’s reagent, 5-5′-Dithiobis(2-nitrobenzoic acid) (DTNB) [33]. The thiol concentration was estimated according to the method described previously [34].

Loin samples (1.5 g) were homogenized (11,600 rpm, 30 s, with ice cooling) with 12.5 mL of 2-(N-morpholino) ethanesulfonic acid buffer (MES) 0.05 M (pH 5.8).

Two hundred and fifty microliters of the homogenate or digest was treated with 750 µL of 5% sodium dodecyl sulphate (SDS) in 0.10 M tris(hydroxymethyl)-aminomethane (TRIS) buffer (pH 8.0). The samples (homogenate or digest) were heated at 80 °C for 30 min, centrifuged (3000× *g*, 20 min), and the supernatants were filtered (0.45 µm) for further analysis.

Thiol concentration was determined by mixing 50 µL of supernatants, 200 µL of 5% SDS/TRIS buffer (pH 8.0), and 50 μL of 10 mM DTNB dissolved in 0.10 M TRIS buffer (pH 8.0). The absorbance at 412 nm was measured before the addition of DTNB reagent (ABS_412 nm before_) and 30 min after adding the DTNB reagent (ABS_412nm after_) protected against the light. Thiol concentration was quantified using a standard curve of L-cysteine (1000–32.25 µM) diluted in 5% SDS/TRIS buffer (pH 8.0). The absorbance corresponding to thiol concentration in each sample was calculated as follows (Equation (2)):

Corr_Abs412_ = ABS _412nm after_ − ABS _412nm before_ − ABS _412 nm blank_ (2)

The protein concentration was determined by a Thermo Scientific Pierce Rapid Gold BCA Protein Assay Kit, measured spectrophotometrically at 480 nm and quantified using a standard curve of BSA (2.5–0.3125 g/L) diluted in 5% SDS/TRIS buffer (pH 8.0). Absorbance readings were measured using a microplate-reader spectrophotometer. Assays were performed in duplicate. Results were expressed as nmol Cys/mg protein.

### 2.6. Statistical Analysis

Statistical analysis of the data was performed using SPSS statistics version 22 [35]. A one-way variance analysis was performed to evaluate the effect of the nitrate/nitrite in dry-cured loin, followed by Tukey’s HSD post hoc test. Differences were considered significant at *p* ≤ 0.05. Results are presented as mean ± standard deviation.

## 3. Results

### 3.1. Fatty Acid Composition, Protein and Lipid Oxidation Product Contents of Dry-Cured Loins Used in the In Vitro Digestion

Table 2 shows the fatty acid composition of dry-cured loins used in the in vitro digestion. The oleic acid (C18:1 n-9) was by far the most predominant fatty acid in dry-cured Iberian loins, accounting for 44.70–48.46 g/100 g of total FAME. The most abundant saturated fatty acid was palmitic acid (C16:0), followed by stearic acid (C18:0), which represented 35.75–38.33% of the total fatty acids. The amount of NO_2_Na/NO_3_K in the formulation of dry-cured loins did not have a significant effect on the contents of individual fatty acids. Higher amounts, but not to a significant extent, of polyunsaturated fatty acids (C18:2, n-6, C18:3 n-3, and C20:4 n-6) were found in 75 and 150 mg/kg NOx dry-cured loins compared to 0 and 37.5 mg/kg NOx counterparts.

Dry-cured loin showed significant differences in the initial contents of protein (thiol and carbonyls) and lipid oxidation (conjugated dienes and malondialdehyde) products, residual nitrite, and non-protein nitrogen as affected by the ingoing amounts of nitrate/nitrite (Table 3).

Initial lipid oxidation markers in dry-cured loins showed higher oxidation in uncured dry-cured loins (0 mg/kg NOx) and those with the low nitrate/nitrite treatment (37.5 mg/kg NOx). Thus, compared to uncured dry-cured loins, nitrate/nitrite dry-cured loins had significantly fewer amounts of conjugated dienes. Similarly, dry-cured loins formulated with 75 and 150 mg/kg NOx had lower MDA contents, but not to a significant extent, than those elaborated without and with the lower dose of nitrate/nitrite (37.5 mg/kg NOx).

Protein oxidation marker contents were significantly different among dry-cured loin batches. Hence, thiol contents were higher in loins formulated with nitrate/nitrite (37.5, 75, and 150 mg/kg NOx) than in non-NOx added equivalents (0 mg/kg NOx). Thiol contents in dry-cured loins formulated with 37.5 and 75 mg/kg NOx had significantly higher thiol values than counterparts without added nitrate/nitrite. Protein carbonyl contents did not significantly differ among dry-cured loins with 0, 75, and 150 mg/kg NOx added. On the other hand, the loin samples formulated with the lower nitrate/nitrite dose (37.5 mg/kg NOx) had significantly lower carbonyl contents than those from loins with 75 mg/kg NOx added.

Nitrite curing of loins (37.5, 75, and 150 mg/kg NOx) significantly increased the content of NOMb in dry-cured loins compared with dry-cured loins without NOx added. In these samples, the formation of ZnPP was also detected, but not in loin samples with nitrite added. Regarding non-heme iron contents, the NHI content was significantly higher in uncured loins than in the cured ones. The NHI content was significantly lower in the loins with 150 mg/kg NOx than in 37.5 and 75 mg/kg NOx.

### 3.2. Residual Nitrite Content in Dry-Cured Loins and the Digests

**Figure 2:**
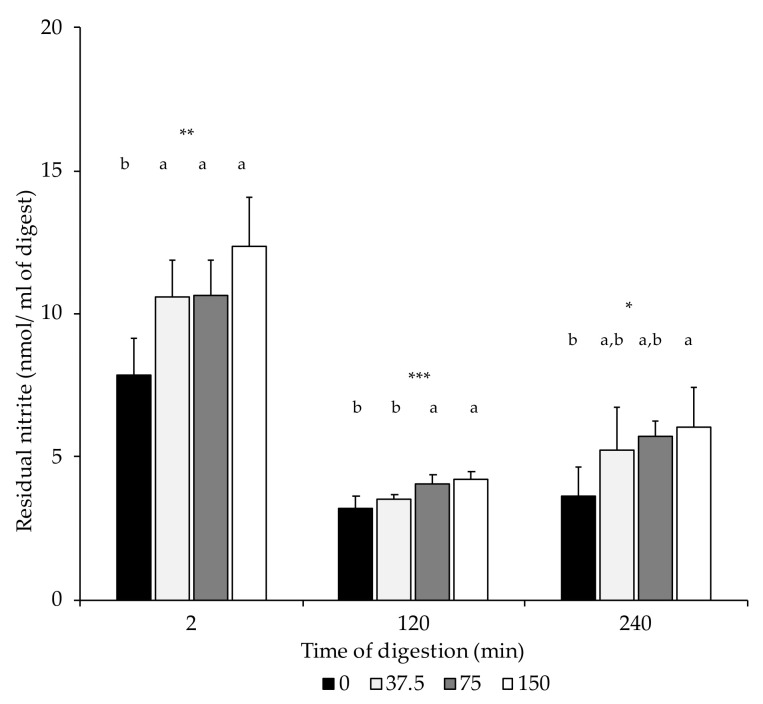
Nitrite content (nmol/mL of digest) in digest at the end of the oral (2 min), gastric (120 min), and intestinal phase (240 min). n.s.: not significant; *: *p* ≤ 0.05; **: *p* ≤ 0.01; ***: *p* ≤ 0.001. a,b: Values for the same time of sampling bearing different letters are significantly different at *p* ≤ 0.05, Tukey’s test.

In this study, the initial residual nitrite (dry-cured loin) was present in low amounts compared to the ingoing dose (0, 37.5, 75, and 150 mg/kg NOx). Loins formulated with 150 mg/kg NOx had significantly higher residual nitrite content than the non-NOx-added equivalents (Table 3). At the end of the oral (2 min), gastric (120 min), and intestinal phase (240 min), residual nitrite contents in digests of loins without nitrate/nitrite added (0 mg/kg NOx) were lower than in the NOx-added digest equivalents, although differences were only statistically significant in the 150 NOx group (Figure 2).

### 3.3. Non-Protein Nitrogen Content in Dry-Cured Loins and the Digests

**Figure 3:**
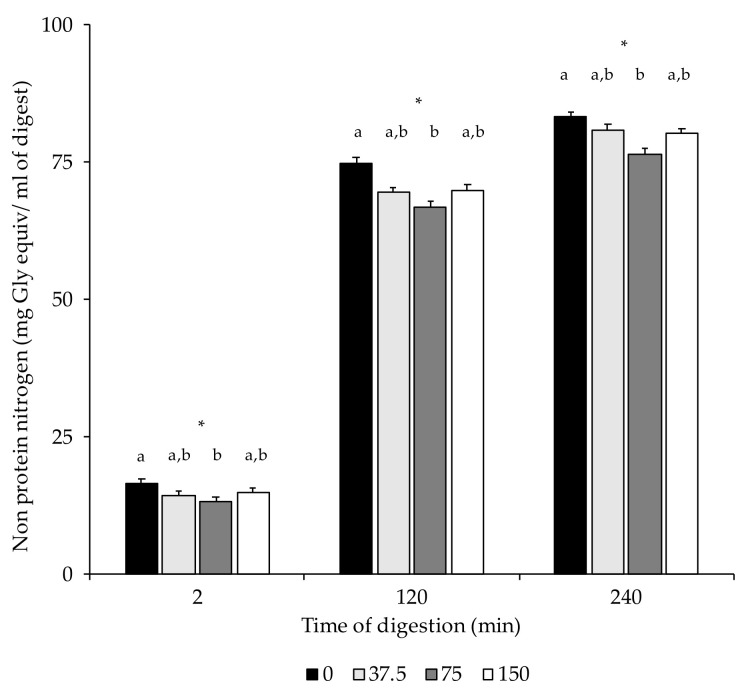
Non-protein nitrogen content (mg Gly equiv/mL of digest) in digest at the end of the oral (2 min), gastric (120 min), and intestinal phase (240 min). *: *p* < 0.05. a,b: Values for the same time of sampling bearing different letters are significantly different at *p* ≤ 0.05, Tukey’s test.

In the present study, NPN contents were only significantly higher in 0 mg/kg NOx dry-cured loins than in 75 NOx counterparts (Table 3). Figure 3 shows the non-protein nitrogen contents in the dry-cured loin digests at the end of the in vitro oral (2 min), gastric (120 min), and intestinal phase (240 min). Due to digestion, higher NPN concentrations were measured in the gastric and intestinal digests compared to the digests in the oral phase. In the three stages, 0 NOx loin digests had higher NPN contents than those with 75 mg/kg NOx added. No differences in proteolysis, measured as NPN, were found among digests of loins formulated with different ingoing amounts of nitrate/nitrite.

### 3.4. Lipid Oxidation Products: Conjugated Dienes and Malondialdehyde Concentration in Dry-Cured Loins during In Vitro Digestion

**Figure 4:**
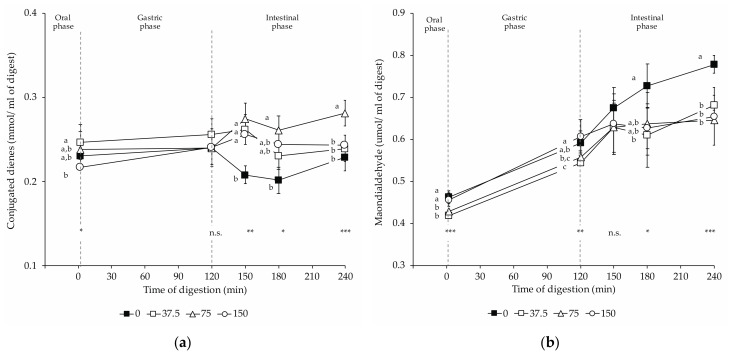
Changes in lipid oxidation products in dry-cured loins formulated with different amounts of NO_2_Na/NO_3_K (0, 37.5, 75, and 150 mg/kg) during in vitro gastrointestinal digestion: (**a**) conjugated dienes; (**b**) malondialdehyde. The time 2 min represents the end of the oral phase, time 120 min, the end of the gastric phase; meanwhile, times 150 and 180 min represent intermediate times of the intestinal phase that ends at time 240 min. *: *p* < 0.05; **: *p* < 0.01; ***: *p* < 0.001. a,b,c: Means for the same time of sampling with different letters are significantly different (*p* ≤ 0.05, Tukey’s test).

Figure 4 shows that the progress of lipid peroxidation was followed by measuring the concentration of conjugated dienes and MDA in the digests.

Conjugated diene contents revealed slight changes during in vitro digestions (Figure 4a). Upon oral digestion, 37.5 NOx digests contained significantly more conjugated dienes than 150 NOx digests. In 0 NOx loin digests, lower amounts of conjugated dienes were found during intestinal digestions than in digests from loins with nitrate/nitrite added. During duodenal digestion, 75 NOx digests resulted in significantly higher concentrations of conjugated dienes compared to 0 NOx ones. At the end of the intestinal stage, 75 NOx digests had the highest conjugated diene content, and no significant differences were observed between the other digests.

In the present study, an overall increase in MDA concentration occurred from the oral to the intestinal in vitro digestion with significant differences in MDA concentration among experimental batches. The MDA concentration in the digest in the oral phase showed significant differences among groups (Figure 4b), although of small magnitude. While in 150 NOx, digests were found with significantly higher MDA than in 37.5 NOx digests, the other two experimental groups exhibited intermediate contents that did not show significant differences to former groups. Compared to non-NOx digests, nitrate/nitrite-curing led to significantly lower MDA concentrations during the intestinal phase (180 and 240 min). For NOx dry-cured loin digests, no significant differences were noticed at the end of the intestinal digestion phase.

### 3.5. Protein Oxidation Products: Protein Carbonyl and Thiol Contents in Dry-Cured Loins during In Vitro Digestion

**Figure 5:**
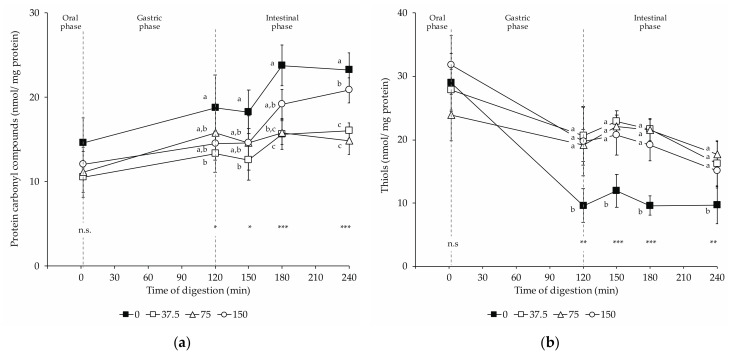
Changes in protein oxidation products in dry-cured loins formulated with different amounts of NO_2_Na/NO_3_K (0, 37.5, 75, and 150 mg/kg) during in vitro gastrointestinal digestion: (**a**) protein carbonyls; (**b**) thiols. The time 2 min represents the end of the oral phase, time 120 min, the end of the gastric phase; meanwhile, times 150 and 180 min represent intermediate times of the intestinal phase that ends at time 240 min. *: *p* < 0.05; **: *p* < 0.01; ***: *p* < 0.001. a,b: Means for the same time of sampling with different letters are significantly different (*p* ≤ 0.05, Tukey’s test).

The oxidation of proteins was monitored by determining the formation of protein carbonyls and the loss of thiols. Figure 5 shows (a) the protein carbonyl compound con-centration and (b) the thiol concentration in digests of dry-cured loins formulated with different amounts of nitrate/nitrite (0, 37.5, 75, and 150 mg/kg NOx) during in vitro digestion.

Protein carbonyls increased throughout in vitro digestion of dry-cured loins (Figure 5a). At the end of the oral step, no significant differences were found in the carbonyl contents among groups. In return, the amounts of carbonyls varied among the digests of loins with different levels of nitrate/nitrite upon the gastric and intestinal digestion. Digests of non-NOx loins had increased carbonyl contents after the gastric and throughout the intestinal phase. Thus, the carbonyl content was significantly higher in 0 NOx loins digests than in 37.5 NOx digests. Otherwise, a definite effect of NOx dose on the promotion of protein carbonylation was observed. Thus, the carbonyl levels in the 75 and 150 NOx digests were intermediate to those found in the 0 and 37.5 NOx digests. Therefore, in these digests (75 and 150 NOx), the carbonyl contents were lower than in 0 NOx digests, but not to a significant extent. At the end of the intestinal phase, the significantly highest carbonyl contents were measured in the 0 NOx-added loin digests, followed by the 150 mg/kg and then by 75 and 37.5 NOx digests.

Simulated digestion affected the content of thiols in all groups of dry-cured loins, being detected a loss in the thiol contents in digests upon gastric and intestinal digestions (Figure 5b). Upon the gastric and during the intestinal phase, nitrite-curing resulted in a higher concentration of thiols when compared to 0 NOx equivalents. In these phases, no differences were found in the thiol concentration among the digests of dry-cured loins with decreasing amounts of nitrate/nitrite (37.5, 75, and 150 mg/kg NOx).

## 4. Discussion

The present study, using dry-cured loins with decreasing amounts of nitrate/nitrite, allowed us to evaluate the effect of added curing salts on lipid and protein oxidation during the simulated digestion of this kind of traditional dry-cured product.

Nitrite exerted an antioxidant effect in both dry-cured loins and digests before and after simulated digestion. As expected, nitrate/nitrite addition reduced the intensity of lipid and protein oxidative reactions, while its removal enhanced the formation of oxidation products from lipids and proteins in both dry-cured loins and digests.

Initial oxidative status in dry-cured loins reflected the marked effect of nitrite controlling lipid and protein oxidation that led to lower amounts of conjugated dienes, MDA and carbonyls, and higher thiol contents in nitrate/nitrite dry-cured loin batches. Similar results previously described the effect of nitrite as an antioxidant in dry-cured meat products and in which nitrite addition significantly lowered lipid and protein oxidative phenomena [36,37,38].

In dry-cured loins formulated with nitrate/nitrite, low traces of nitrite were found, turning out that nitrite is a highly reactive molecule that generates nitrosylmyoglobin by reaction with myoglobin [2]. In this sense, the NOMb content was higher the greater the concentration of NOx used in the formulation of the products. Nitrosylmyoglobin formation was also detected in those dry-cured loin samples formulated without NOx, although at low concentrations. Zinc porphyrin was detected in 0 NOx loins, while it was not present in the cured samples, being consistent with previous results [39,40]. Chen et al. [41] described that nitrite curing led to less destruction of the stable NO-heme by stabilizing the porphyrin ring in the NOMb molecule, thereby inhibiting Fe^2+^ release. Free Fe^2+^ catalyzes the Fenton reaction through which oxidative processes are initiated [42]. Considering the amounts of NOMb in dry-cured loins formulated with different amounts of nitrate/nitrite, 0 NOx loins contained less than 60% of myoglobin in the NOMb chemical form. It might imply a higher release of Fe^2+^ from myoglobin under drying or simulated digestion conditions to initiate oxidation reactions. In the present study, the NHI contents were higher the lower the nitrate/nitrite added to dry-cured loins and the NOMb contents in samples.

During and after gastrointestinal digestion, conjugated dienes slightly augmented while concentrations of TBARS had a significant increase, confirming the ingoing lipid oxidation during digestion reported by other authors. The oxidative process, initiated during drying, can then develop during digestion. Indeed, the physicochemical conditions under the simulated gastric phase, such as oxygen pressure and low pH, favor the propagation of oxidation [16,17,18]. Lipid peroxidation is generally assessed by measuring the major initial peroxidation products, such as conjugated dienes and lipid hydroperoxides, and minor breakdown products, including some aldehydic compounds such as MDA, saturated and unsaturated aldehydes, 4-hydroxy-2-alkenals, and hydrocarbons.

In the present work, conjugated dienes and MDA were assayed to evaluate primary and lipid oxidation end products, respectively. Conjugated dienes are formed by an initial rearrangement of the double bonds of polyunsaturated fatty acids due to radical chain reactions. Upon reaction with oxygen, lipid hydroperoxides are produced, which readily decompose into various other products, including MDA. In our experiment, changes in conjugated diene concentrations were distinct in uncured and cured loin digests after simulated digestion compared to initial values before digestion, in which moderate increases of conjugated dienes and MDA were noted during gastric and duodenal simulated digestion phases.

Increased oxidation during digestion of dry-cured loins under in vitro conditions was confirmed in our study using TBARS assay. The formation of MDA initiated in the gastric phase boosted during the intestinal phase to reach the highest values at the end of the intestinal phase. The rise in TBARS until duodenal digestion has been reported previously by other authors [16,18,43]. Additionally to the favorable conditions for oxidation during the gastric phase, the digestive tract is characterized by the enzymatic degradation of lipids and proteins, rendering fatty acids and amino acids more sensitive to free radical attack [44,45].

The lower MDA concentration, especially in the intestinal phase, of nitrite-cured loin digests compared to uncured equivalents reflects the antioxidant activity of nitrite even during in vitro gastrointestinal digestion, being consistent with recently published works [16]. This fact is attributable to the antioxidant mechanisms of nitrite that include i. the antioxidant activity of the formed NOFe(II)Mb, ii. the nitric oxide ferrous complexes and S-nitroso-cysteine, and iii. the inhibition of the Fenton reaction [46]. Even though nitrite has a marked antioxidant effect, nitrite could exert both pro-oxidant and antioxidant effects depending on its ratio to the reactive oxygen substances. Thus, a 1:1 proportion of ·NO to ROS can induce lipid peroxidation by peroxynitrite (ONOO-) formation, while an excess of ·NO results in inhibition [16,47]. Our results show that a reduction of 75% in ingoing amounts of nitrate/nitrite (37.5 mg/kg) mitigates the formation of lipid oxidation products during simulated digestion of cured compared to uncured loin digests.

Although the objective of nitrite removal from cured dry loin is to lessen the potential formation of nitrosamines during digestion, the findings showed adverse results derived from its removal. Thus, the higher lipid oxidation, especially the higher MDA content, in non-cured loin digests has significant relevance compared to the different behavior found in the NOx-added loin digests. In the small intestine, MDA, a recognized toxic and potentially carcinogenic compound, is absorbed into the bloodstream and transported to several organs in which free MDA could damage DNA by the formation of the MDA–DNA adduct pyrimido [1,2-α] purine-10(3H)-one-2′-deoxyribose (M1dG) [10,48]. Furthermore, oxidative stress can also activate nitrosamines, leading to the formation of α-hydroxy nitrosamines, which spontaneously decompose to form an alkyl diazonium ion and a free alkyl carbocation, able to alkylate DNA, as was reported previously [49]. Under simulated digestion, the highly reactive ROS cause oxidative damage to proteins [50].

As previously reported for the formation of LOP-derived compounds, as simulated digestion progresses, the protein oxidation increased for all digests. In the present study, the increment of the carbonyl concentration together with the decrease in thiol content agrees with previously reported findings [51]. Accordingly to Zhang et al., [50], the generation of carbonyl compounds is the most abundant manifestation of protein oxidation along with the loss of free thiol groups. Different pathways mediate in the protein oxidation, such as (1) metal-catalyzed oxidation, such as Fe^2+^, in which carbonyls are formed in the side chain of arginine, lysine, proline, and threonine, (2) protein carbonylation formation via the glycation of lysine residues due to reducing sugars, or (3) protein carbonylation due to lipid oxidation products, such as MDA and hydroxy-alkenals, which can form adducts with proteins [50]. In this regard, many studies have found correlations between the progression of the oxidation of lipids and proteins in model systems and meat products, so it is reasonable to assume the interaction between the oxidation of lipids and proteins by transferring radical species between both phenomena [45,50,52].

During in vitro gastrointestinal digestion, the formation of protein carbonyls was inhibited by nitrite used in the curing of loins, which is in agreement with recently published works by Van Hecke et al. [16,17] The high content of non-heme iron and MDA and the low concentration of the more stable NOMb in uncured loin digests make their proteins more prone to carbonylation, as occurred during in vitro digestion. Due to the low amounts of residual nitrite and the absence of differences in its content between dry-cured and uncured loin digests, the results indicate that the greater intensity of protein carbonylation in uncured loin digests is due to the NHI, MDA, and NOMb contents related to the antioxidant effect of nitrite. A dose-dependent effect of nitrite on carbonylation of proteins during digestion could explain the significant upward trend in the concentration of carbonyls in 150 mg/kg NOx dry-cured loin digests compared with intermediate concentrations of NOx (37.5 and 75 mg/kg) counterparts.

Previously, several authors suggested that MDA may react with amino acid side chains in proteins, contributing to protein aggregation and decreasing susceptibility to pepsin activity, potentially resulting in lower protein digestibility and leading to abnormal fermentation in the colon altered with the formation of toxic and carcinogenic compounds [51,53,54]. In this regard, this negative effect was not detected either in the gastric or intestinal phases as a result of the increased protein oxidation in the uncured loin digests compared to the homologous cured loin digests. The NNP values showed increases throughout mimicked digestion, and the differences among experimental groups maintained the initial differences found in dry-cured loins. Discrepancies with previous papers could be related to the different experimental samples (dry-cured loin, beef patties, myofibrillar proteins, etc.) used in the distinct experiments.

The thiol group on the cysteine residue is highly reactive and sensitive to oxidative damage in the presence of ROS during in vitro digestion, being that thiol oxidation is the most abundant protein oxidation mechanism [51]. In the present study, a significant decrease in free thiols was observed during in vitro digestion. This thiol loss could be attributed to direct ROS attack at thiol groups on the cysteine residue in meat proteins, converting them to disulfide bonds and other thiol oxidation products (sulfenic, sulfinic, and sulfonic acid, and thiosulfinates) [50]. According to our results, Hu et al. [55], in simulated digested roasted fish, described that the content of free thiols increased after gastric digestion, and, at the end of intestinal digestion, thiol levels decreased below the initial concentration. These authors concluded that the rise and fall of thiols was probably due to the release of sulfhydryl groups by the enzymatic hydrolysis of the proteins in the gastric stage and subsequently oxidized during intestinal digestion. This theory could explain the increase in thiol concentration after 150 min of digestion. In uncured loin digests, the loss of thiol groups occurs intensely during the gastric phase and continues moderately during the intestinal phase. This same pattern occurred during the digestion of loins cured with different amounts of ingoing nitrate/nitrite, but the losses of thiol groups are significantly lower. The removal of nitrifying agents causes the loss of thiol groups due to oxidative phenomena, while in cured loin digests, the loss of thiols is controlled even in the case of 75% reduction of the maximum doses used. Therefore, it seems unlikely that the added nitrate/nitrite decreased thiol oxidation after in vitro gastrointestinal digestion.

A high concentration of carbonyls leads to a decrease in proteolysis by decreasing the susceptibility of oxidized proteins to pepsin. This fact causes an increase in the amount of protein reaching the colon, susceptible to be fermented by the colonic microbiota [17,18,51]. Fermented protein results in the formation of potentially toxic products, such as ammonia, phenol, p-cresol, and indole, but a clear association between a higher concentration of these compounds and enhanced colon cancer promotion in rats has not been demonstrated [56]. Additionally, previous studies described that nitrite-cured meats did not increase the formation of the NOC-derivative DNA adduct O^6^-C-MeG during colonic fermentation, while high concentrations of heme iron and or fat do in the colonic phase of digestion [16,17].

## 5. Conclusions

To the best of our knowledge, this study was the first to study the changes in lipid and protein oxidation phenomena of dry-cured loin with reduced ingoing nitrate/nitrite under mimetic gastrointestinal conditions. Our results indicate that the removal of nitrate/nitrite in dry-cured loins favors the formation of lipid and protein oxidation products during in vitro digestion. Initial oxidation values generated during the dry-curing process, in which lipids and proteins oxidized at a different intensity depending on the amounts of curing salt used, affect the subsequent formation of compounds such as MDA, carbonyls, and thiols during the enzymatic digestion. The removal of nitrites to obtain a healthy meat product by minimizing the possible formation of nitrosamines is affected by the increase in oxidative phenomena that lead to higher amounts of primary and secondary lipid peroxidation metabolites and products from protein carbonylation. The levels of 37.5 mg/kg nitrate/nitrite added for technological reasons in the formulation of cured loins prevent the formation of lipid peroxidation products and carbonyls from protein oxidation and the loss of thiols and might avoid the formation of NOCs during digestion. The formation of toxic compounds, such a NOCs, should be evaluated in future experiments.

## Figures and Tables

**Table 1:** Composition of simulated digestive fluids used for the in vitro digestion.

			SSF pH 7.0	SGF pH 3.0	SIF pH 7.0
	Conc. in Stock		Conc. in SSF	Conc. In SGF	Conc. in SIF
	g/L	mol/L	mmol/L	mmol/L	mmol/L
KCl	37.3	0.5	15.1	6.9	6.8
KH_2_PO_4_	68	0.5	3.7	0.9	0.8
NaHCO_3_	84	1	13.6	25	85
NaCl	117	2	—	47.2	38.4
MgCl_2_(H2O)_6_	30.5	0.15	0.15	0.1	0.33
(NH_4_)_2_CO_3_	48	0.5	0.06	0.5	—
For pH adjust					
NaOH		1.0			
HCl		6.0	1.1	15.6	8.4
CaCl_2_ (H_2_O)_2_	44.1	0.3	1.5 (0.075 *)	0.15 (0.075 *)	0.6 (0.3 *)
Salivary amylase			75 U/mL	—	—
Pepsin			—	2000 U/mL	—
Pancreatin			—	—	100 U/mL **
Bile			—	—	10 mM

* Ca^2+^ concentration in the final digestion mixture. ** Sufficient pancreatin to provide 100 U/mL of trypsin (TAME Units).

**Table 2:** Fatty acid composition (g/100 g total FAMEs) of dry-cured loins used in the in vitro digestion.

	NO_2_Na/NO_3_K (mg/kg) in Formulation				
	0	37.5	75	150	*p*
C 14:0	1.46 ± 0.06	1.42 ± 0.08	1.37 ± 0.12	1.54 ± 0.57	0.827
C 16:0	26.82 ± 0.80	26.30 ± 0.50	25.17 ± 3.18	26.22 ± 1.37	0.541
C 16:1 n-7 *t*	0.20 ± 0.04	0.20 ± 0.04	0.21 ± 0.04	0.21 ± 0.05	0.928
C 16:1 n-7 *c*	4.40 ± 0.93	4.23 ± 0.45	4.65 ± 1.58	4.72 ± 2.06	0.938
C 17:0	0.19 ± 0.06	0.20 ± 0.09	0.19 ± 0.06	0.22 ± 0.09	0.883
C 17:1	0.24 ± 0.06	0.26 ± 0.10	0.27 ± 0.10	0.27 ± 0.08	0.907
C 18:0	11.11 ± 1.34	10.74 ± 0.34	10.58 ± 1.61	12.11 ± 2.66	0.505
C 18:1 n-9 *t*	0.14 ± 0.02	0.14 ± 0.03	0.16 ± 0.01	0.18 ± 0.04	0.134
C 18:1 n-9 *c*	47.00 ± 1.13	48.06 ± 0.63	48.46 ± 1.96	44.70 ± 8.66	0.563
C 18:1 n-11 *c*	4.94 ± 0.69	4.97 ± 0.31	4.84 ± 1.51	5.55 ± 1.98	0.821
C 18:2 n-6 *c*	2.52 ± 0.68	2.43 ± 0.41	2.99 ± 0.28	2.95 ± 0.46	0.193
C 20:0	0.13 ± 0.03	0.14 ± 0.02	0.14 ± 0.01	0.16 ± 0.04	0.515
C 18:3 n-3 *c*	0.11 ± 0.04	0.10 ± 0.02	0.12 ± 0.02	0.12 ± 0.02	0.504
C 20:1 n-9 *c*	0.62 ± 0.20	0.71 ± 0.16	0.71 ± 0.09	0.83 ± 0.22	0.355
C 20:4 n-6 *c*	0.12 ± 0.03	0.11 ± 0.02	0.14 ± 0.03	0.22 ± 0.15	0.177

**Table 3:** Conjugated dienes, malondialdehyde, carbonyls, thiols, residual nitrite, non-nitrogen protein, NOMb, ZnPP, and non-heme iron in dry-cured loins used in the in vitro gastrointestinal digestion.

	NO_2_Na/NO_3_K (mg/kg) in Formulation				
	0	37.5	75	150	*p*
Lipid and protein oxidation indicators					
Conjugated dienes ^1^	2.7 a ± 0.17	2.5 b ± 0.17	2.4 b ± 0.08	2.4 b ± 0.13	0.005
Malondialdehyde ^1^	0.8 ± 0.19	0.8 ± 0.13	0.6 ± 0.05	0.6 ± 0.02	0.035
Carbonyls ^2^	17.4 a,b ± 1.57	15.5 b ± 0.89	20.0 a ± 3.71	19.4 a,b ± 1.51	0.021
Thiols ^2^	35.7 b ± 4.43	49.3 a ± 6.82	49.5 a ± 5.16	43.6 a,b ± 2.93	0.001
NOMb/ZnPP and non-heme Fe contents					
NOMb ^3^	1.1 b ± 0.63	2.4 a ± 0.44	2.6 a ± 0.45	3.0 a ± 0.61	0.001
ZnPP ^3^	0.9 ± 0.43	n.d.	n.d.	n.d.	0.001
Non-heme Fe ^3^	0.081 a ± 0.006	0.048 b ± 0.03	0.048 b ± 0.003	0.042 c ± 0.004	0.001
Residual nitrite and non-protein nitrogen contents					
Nitrite ^3^	0.1 b ± 0.03	0.2 a,b ± 0.08	0.1 a,b ± 0.07	0.2 a ± 0.06	0.015
NPN ^4^	2.6 a ± 0.10	2.2 a,b ± 0.23	2.1 b ± 0.50	2.6 a,b ± 0.12	0.022

a,b,c: Means bearing different letters are significantly different at *p* ≤ 0.05, Tukey’s test. n.d.: Not detected. ^1^ µmol/100 g; ^2^ nmol/mg protein; ^3^ mg/100 g, ^4^ g Gly equiv/100 g.

## Data Availability

The data that support the findings of this study are openly available in Mendeley Data at http://doi.org/10.17632/pswpb9wtxy.1, (accessed on 29 July 2021).
